# Quantifying the quantum nature of high-spin YSR excitations in transverse magnetic field

**DOI:** 10.1126/sciadv.adq0965

**Published:** 2024-10-18

**Authors:** Niels P. E. van Mullekom, Benjamin Verlhac, Werner M. J. van Weerdenburg, Hermann Osterhage, Manuel Steinbrecher, Katharina J. Franke, Alexander A. Khajetoorians

**Affiliations:** ^1^ Institute for Molecules and Materials, Radboud University, Nijmegen, Netherlands.; ^2^ Fachbereich Physik, Freie Universität Berlin, Berlin, Germany.

## Abstract

Excitations of individual and coupled spins on superconductors provide a platform to study quantum spin impurity models as well as a pathway toward realizing topological quantum computing. Here, we characterize, using ultralow temperature scanning tunneling microscopy/spectroscopy, the Yu-Shiba-Rusinov (YSR) states of individual manganese phthalocyanine molecules with high spin on an ultrathin lead film in variable transverse magnetic field. We observe two types of YSR excitations, depending on the adsorption geometry. Using a zero-bandwidth model, we detail the role of the magnetic anisotropy, spin-spin exchange, and Kondo exchange. We illustrate that one molecular type can be treated as an individual spin, whereas the other type is best described by a coupled spin system. Using the field dependence of the YSR excitations combined with modeling, we describe the quantum phase of each excitation type. These results provide an insight into the quantum nature of YSR excitations in magnetic field and a platform to study spin impurity models on superconductors in magnetic field.

## Teaser

The YSR excitations of manganese phthalocyanine molecules are studied in a transverse magnetic field revealing a complex response.

## INTRODUCTION

An individual spin impurity exchange coupled with a superconductor can lead to local in-gap excitations referred to as Yu-Shiba-Rusinov (YSR) excitations (*1*–*3*). These in-gap excitations define the energy difference between binding or unbinding a quasiparticle to the spin impurity. The excitation energy depends primarily on the competition between the superconducting pairing energy (Δ) and the Kondo exchange energy (*J*_K_) and defines two distinct regimes given by a bound or unbound quasiparticle. The transition between these two regimes is often referred to as the quantum phase transition (QPT). Experiments based on scanning tunneling microscopy/spectroscopy (STM/STS) have been widely successful at studying YSR excitations derived from atomic and molecular impurities (*4*–*6*). Nevertheless, most experiments were performed in the absence of a magnetic field. Yet, methods that involve magnetic field are essential to determine the parity of the quantum states involved in the excitation, namely the “excitation pathways,” as well as the spin state of the impurity. In contrast to high-spin impurities, for *S* = 1/2, the excitation pathway as a function of magnetic field simplifies (*7*, *8*). This is because multiple interactions, such as interatomic exchange, competing Kondo exchange energies, and single-ion magnetic anisotropy, can be neglected. Yet, a vast majority of spin impurities on surfaces are derived from 3*d* or 4*f* atomic spins (*9*–*11*), where the total spin *S* > 1/2 necessitates consideration of competing energy scales on the excitation pathways.

Going beyond the *S* = 1/2 picture necessitates a quantum description that considers multiple energy scales, such as the Kondo exchange coupling in a number of channels (i.e., *J*_K*_i_*_), intra-atomic exchange (e.g., Hund), on-site Coulomb energies (e.g., *U*), and single-ion magnetic anisotropy (*12*). Spin impurity models are the most prevalent way to quantify the role of various interactions on the resultant excitation pathways (*13*–*15*). Most often, the description is reduced to a giant spin model, neglecting Hund’s exchange and the interplay of Coulomb interactions, and this giant spin is coupled to a bath. On a superconductor, the latter is often treated in a zero-bandwidth model which shows that the role of renormalization (*16*) is rather weak, thus further reducing the computational complexity of the problem. On the basis of these model predictions, the excitation pathways of high-spin impurities is determined by a sensitive interplay between multiple energy scales, which can only be discriminated by systematically modifying an energy scale, for instance, by a magnetic field (*13*, *14*). Certain experimental approaches have been used to modify the Kondo exchange energy in a limited range, enabling identification of the excitation pathways (*17*–*19*). While an applied magnetic field would be an ideal perturbation (*7*, *8*), the upper critical field of typical Bardeen-Cooper-Schrieffer (BCS) superconductors used in experiments corresponds to an energy much smaller than the desired Zeeman energies needed to observe changes in the YSR excitations.

Here, we quantify the response of YSR excitations of high-spin impurities to a large transverse magnetic field. We start by depositing individual manganese phthalocyanine (MnPc) molecules on the surface of a quantum-confined and superconducting lead (Pb) film. MnPc molecules exhibit two distinct types of YSR excitations, depending on the binding site and orientation of the molecule with respect to the substrate. Because of the combination of quantum confinement and spin-orbit coupling, we observe negligible changes to the superconducting gap structure in response to magnetic fields parallel to the surface, up to *B*_∥_ ≤ 4 T. Using the robustness of the superconductor, we quantify the changes of all the YSR excitations for each molecule type in the presence of an applied transverse magnetic field up to *B*_∥_ = 4 T. Unlike the expected behavior for a *S* = 1/2 impurity, we observe a nonlinear and nonmonotonic evolution of the YSR excitations for both molecule types. We additionally observe multiple YSR excitations and a change of the total number of these excitations in magnetic field. Using a zero-bandwidth spin model, which considers *J*_K*_i_*_, single-ion anisotropy, magnetic exchange, and the Zeeman energy, we quantify the role of these various interactions on the YSR excitations and the excitation pathways. On the basis of this, we identify trends in the model simulations, which reproduce parts of the observed spectra. We also illustrate that the conventional model descriptions fail to capture vital aspects observed in the experiment in an applied magnetic field, suggesting that new theoretical understandings that consider transport-based effects and go beyond the zero-bandwidth picture may be necessary to understand the YSR problem in magnetic field.

## RESULTS

**Fig. 1.**
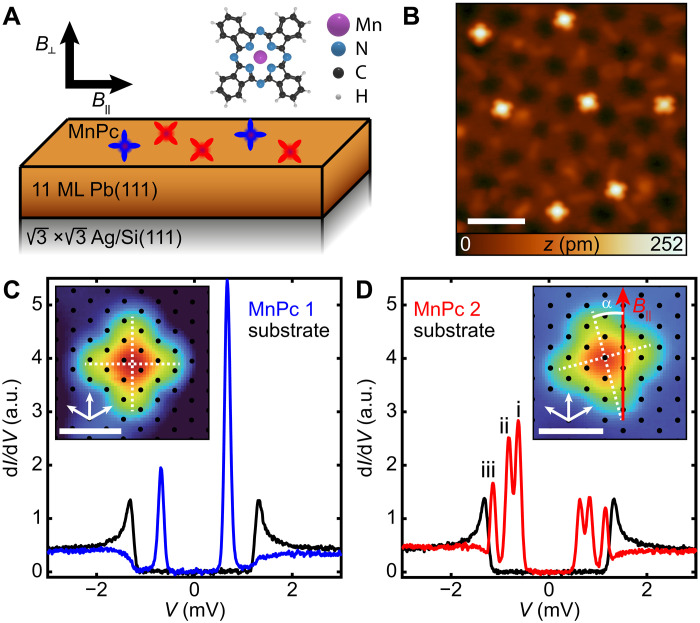
YSR excitations of individual MnPc molecules on the surface of an 11 ML Pb film. (**A**) Schematic of the sample system studied, composed of individual MnPc molecules, an 11 ML Pb film grown on a Si(111)-Ag ($\sqrt{3}\times \sqrt{3}$) surface. (**B**) Constant-current STM image of MnPc deposited on 11 ML of Pb (*V*_S_ = 90 mV, *I*_t_ = 5 pA). Scale bar, 5 nm. (**C**) YSR excitations at *B* = 0 T of a typical MnPc1 type molecule (blue). The substrate spectrum (black) was measured 4 nm away from the molecule. MnPc1 is characterized by having one of its ligand axes parallel to one of the high symmetry directions of the Pb(111) film [inset: MnPc superimposed over the Pb(111) lattice (black), the white dashed lines indicate ligand axes, and white arrows indicate high symmetry directions.] Scale bar, 1 nm. (**D**) YSR excitations at *B* = 0 T of a typical MnPc2 type molecule (red). The substrate spectrum (black) was measured 11 nm away from the molecule. MnPc2 is characterized by having one of its ligand axes bisected by one of the high symmetry directions of the Pb(111) film [inset: similar to (C), the field angle (α) is indicated with respect to the ligand axes] Scale bar, 1 nm. All spectra were measured with *V*_S_ = 6 mV, *I*_t_ = 200 pA, *V*_mod_ = 20 μV, *T* = 30 mK, and a W tip. a.u., arbitrary units.

To study YSR excitations in magnetic field, we started by depositing ultrathin Pb films on the surface reconstruction Si(111)-Ag ($\sqrt{3}\times \sqrt{3}$). A schematic of the experiment is illustrated in Fig. 1A (see Materials and Methods and fig. S1). We intentionally worked with ultrathin Pb films due to their extraordinarily large in-plane upper critical field ($H_{c}^{2}$) (*20*, *21*). We observed a robust hard superconducting gap, which was insensitive to the value of *B*_∥_, up to 4 T (fig. S2). Using these Pb films, we subsequently deposited MnPc onto the surface. Individual MnPc molecules preferentially adsorb on the step edges of the film. By using lateral manipulation with the STM tip (see Materials and Methods), we dragged individual molecules onto chosen locations on a given terrace of the grown Pb film.

We classify two types of YSR spectra at zero magnetic field, as displayed in Fig. 1 (C and D), based on two distinct orientations of the MnPc ligands with respect to the underlying threefold axes of the Pb(111) lattice. We refer to the two types as MnPc1, where one of the ligand axes of the molecule is parallel to one of the high symmetry directions of the Pb(111) film, and MnPc2, where the ligand axes of the molecule are bisected by one of the high symmetry directions of the Pb(111) film (fig. S3). While there are variations in the YSR excitation energies for each MnPc type, we do not find a clear correlation of these variations with the underlying Moiré lattice or a specific binding site (see figs. S3 and S4). We also observe a low-defect density and reproduce the two spectral types for the same molecule that has been positioned on various sites of the film. We note that the interface can be seen through these films, as reported in (*21*), and therefore not all visible defects apparent in the images are necessarily at or near the surface. The two types of spectra correlate with two types of Kondo-like excitations on a larger energy scale [see (*22*) and fig. S6]. We note that we observed a spatial dependence on the intensity of the YSR excitations along the molecule, but all excitations were present at the various probed locations and at the same energies (fig. S5). Therefore, all the subsequent spectra are measured on the center of the molecule.

The YSR excitations of MnPc1 feature one pair of peaks with larger intensity at positive bias voltage, whereas MnPc2 shows three pairs of peaks with larger intensity at negative bias. The inversion in the asymmetry of the YSR excitations between the two types is typically considered attributed to inverting the excitation pathway, i.e., a change of the ground and excited states. We note that within the range of conductances in which the molecule remains stable on the surface, we do not observe a notable change in the YSR excitation energies for both MnPc types (*19*), which would help in identifying the ground state. On bulk Pb(111), both isolated and MnPc molecules in a densely packed monolayer illustrate a three-peak structure (*23*). In that case, the three YSR excitations were ascribed to the excitation of anisotropy-split states from *S* = 1 molecules. The energy and the intensity of the states differed depending on adsorption site. This variation was attributed to variation in *J*_K_. Likewise, Kondo-like resonances have been observed for MnPc on both Pb(111) bulk and Pb thin films (*6*, *22*), which qualitatively agree with the Kondo-like spectra taken on MnPc1 and MnPc2 (fig. S6).

**Fig. 2.**
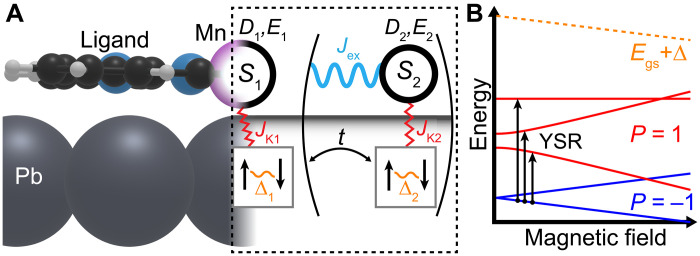
Zero-bandwidth model of the YSR excitations in magnetic field. (**A**) Schematic side view of the MnPc molecule that links the physical system to the spin/superconducting sites in the model (see Materials and Methods). The model considers spin values (*S_i_*), Kondo exchange (*J*_K_i__), spin-spin exchange coupling (*J*_ex_), magnetic anisotropy (*D_i_*, *E_i_*), and hopping between superconducting sites (*t*). (**B**) Example of an energy level diagram for the one site model with *S* = 1, with the ground state (bound quasiparticle, parity *P* = −1) and the excited states (unbound quasiparticle, *P* = 1), easy-axis and transverse anisotropy in a magnetic field, illustrating how this level structure relates to the measured YSR excitations. The orange line indicates the energy of the ground state *E*_gs_ + Δ.

Before exploring the observed magnetic field dependence of both MnPc1 and MnPc2, we describe the expected experimental behavior based on theoretical modeling. We use a zero-bandwidth model with both one (two) superconducting site(s) and one (two) spin site(s), as previously described in (*14*, *24*). Such modeling can capture the interplay between the YSR excitations, including various energy scales, such as exchange couplings and magnetic anisotropy. We additionally consider a Zeeman term and a transverse anisotropy term in the magnetic anisotropy (see Materials and Methods). We do not consider the effect of particle-hole asymmetry, which would lead to different YSR intensities at positive and negative biases, as we are primarily interested in the energy shift of the YSR excitations in response to a magnetic field. Since all excitations are observable at the same energy on both sides of the gap, we capture all the relevant YSR excitations. It has been previously shown that such models capture the qualitative physics, as renormalization effects are typically weak in the YSR problem (*14*, *16*). An example of our modeling is illustrated in Fig. 2B, in which the excitation diagram as a function of the magnetic field can be linked to the measurements. Our choice of considering two spin sites with magnetic anisotropy was motivated by previous ab initio electronic structure calculations of an individual MnPc molecule on Pb(111) (*25*, *26*), aimed at understanding the Kondo behavior in this system (*6*, *22*). On the basis of this input, it was shown that the Mn atom hosts a total spin *S* > 1/2, due to Hund’s exchange, and that there is a notable crystal field splitting. Moreover, in (*26*), it was further shown that the ligands can acquire a spin, which antiferromagnetically couples to the Mn atom. We also consider the effect of a small hopping parameter *t*, in the limit where this does not perturb the superconductivity, as shown in (*24*) for two-impurity systems. The choice to add this hopping parameter was to consider the effect of substrate-mediated hybridization of the two channels, which may occur if one spin is located at the center and the other one at the ligand (*25*, *26*).

On the basis of the zero-bandwidth model and the exploration of the parameter space, we can distinguish three categories of magnetic field–dependent trends: (i) a field-dependent splitting of the YSR excitations that are degenerate at *B* = 0 T. This occurs when the excited state is Kramer’s degenerate, or when the excited state has integer spin without magnetic anisotropy leading to a doubly degenerate state (e.g., ∣±1⟩) that can be accessed via selection rules (fig. S7, A and B). (ii) A nonlinear *B*-dependent evolution of a given YSR excitation. This occurs when rotational symmetry is broken, due to either magnetic anisotropy for *S* > 1/2, or when there is a difference in *g*-factor between two coupled spins (fig. S7, C and D). (iii) A change in the number of YSR excitations for *B*_∥_ ≠ 0 T. A change in the total number of YSR excitations is usually associated with a QPT of the ground state. For *S* > 1/2, there are two types of QPTs to consider. (a) A change in the fermion parity of the ground state, namely a change in the number of bound quasiparticles. This change in parity is always accompanied by a YSR excitation that crosses the gap center (fig. S7, E and F). (b) A parity preserving QPT that changes the ground state spin projection, which may lead to a change in the number of accessible excitations due to selection rules (fig. S7, G and H) (*14*).

**Fig. 3.**
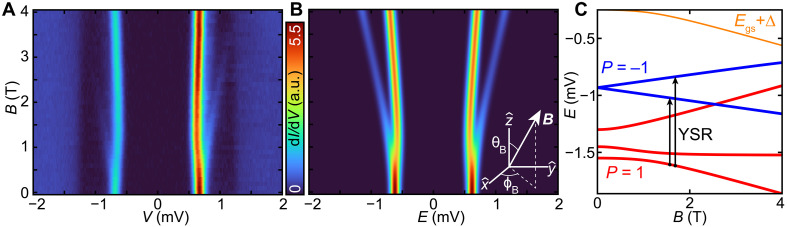
Transverse magnetic field dependence of the YSR excitations of MnPc1 given by a single site. (**A**) A false-color plot of the STS spectra taken at *B*_∥_ in steps of Δ*B*_∥_ = 0.1 T, up to *B*_∥_ = 4 T. All data were measured with *V*_S_ = 6 mV, *I*_t_ = 200 pA, *V*_mod_ = 20 μV, *T* = 30 mK, and a W tip. (**B**) Simulation of the YSR spectra using the one site model with parameters (see Materials and Methods for Hamiltonian): *S* = 1, *g* = 2, *D* = −0.2 mV, *E* = −0.05 mV, Δ = 1.3 mV, *g*_SC_ = 2, *J*_K_ = 0.79 mV, and transverse magnetic field defined using a polar angle θ*_B_* = 90° (from $+\hat{z}$ to $\hat{B}$), and azimuthal angle ϕ*_B_* = 60° (from $+\hat{x}$ to the orthogonal projection of $\hat{B}$ on the *x*-*y* plane). (**C**) Associated Zeeman level diagram of simulated YSR spectra in (B). Energy eigenstates in red (blue) belong to parity *P* = 1 (unbound) [*P* = −1 (bound)]. The orange line indicates the energy of the ground state *E*_gs_ + Δ.

Next, we review the measured YSR excitations of individual MnPc1 molecules as a function of *B*_∥_. In Fig. 3A, we plot the spectra in a high-resolution false color plot with increasing transverse field strength. For the subsequent descriptions, we focus on the subset of YSR excitations at one bias polarity to avoid confusion. For *B*_∥_ < 0.5 T, the sole YSR excitation shows an overall insensitive response to applied magnetic field. Around *B*_∥_ ≈ 0.5 T, the YSR excitation splits asymmetrically, with an asymmetric spectral weight favoring the state closer to the gap center. For *B*_∥_ > 0.5 T, the excitation with higher intensity shows a nonlinear evolution first trending toward the gap center and then after an inflection point moving toward the gap edge. The other split excitation at higher energy shows an almost linear evolution with a different slope from the former state and loses intensity as it approaches the gap edge. We note that none of the excitations crosses the gap center.

The *B*_∥_ evolution of the YSR excitations is a signature of the interplay of a transversal magnetic field and magnetic anisotropy. In Fig. 3B, we illustrate the modeled YSR excitations for a single spin site *S* = 1 with axial and transverse anisotropy, coupled to a single superconductor site in a transverse magnetic field. We note that the modeled YSR excitations are very sensitive to the interplay of the various parameters, and we chose the model parameters that best reproduce the experimental spectra (see fig. S8). We also display the corresponding energy-level diagram (Fig. 3C). This scenario corresponds to an unbound quasiparticle in the ground state, i.e., a free-spin ground state. At *B* = 0 T, the model yields one YSR excitation that corresponds to the transition from the ground state to the Kramer’s degenerate doublet. Because of magnetic anisotropy, the ground state energy is insensitive to *B*_∥_ < 0.5 T. However, the excited state is a spin-½ doublet that is affected by *B*_∥_ resulting in a splitting and the observed two branches. This observed splitting seems to occur at a finite value of *B*_∥_ due to the considered broadening in the calculation (see Materials and Methods). As *B*_∥_ increases, the Zeeman energy becomes larger than the magnetic anisotropy and the ground state aligns with *B*_∥_. This results in a nonlinearity in the *B*_∥_ dependence of the ground state and produces an inflection point. Although the simulation captures the observed trends well, there are other parameter sets that result in a qualitatively similar trend (fig. S8). However, in all the cases we simulated, the best match of parameters always yields a ground state with the same parity, i.e., no bound quasiparticles.

**Fig. 4.**
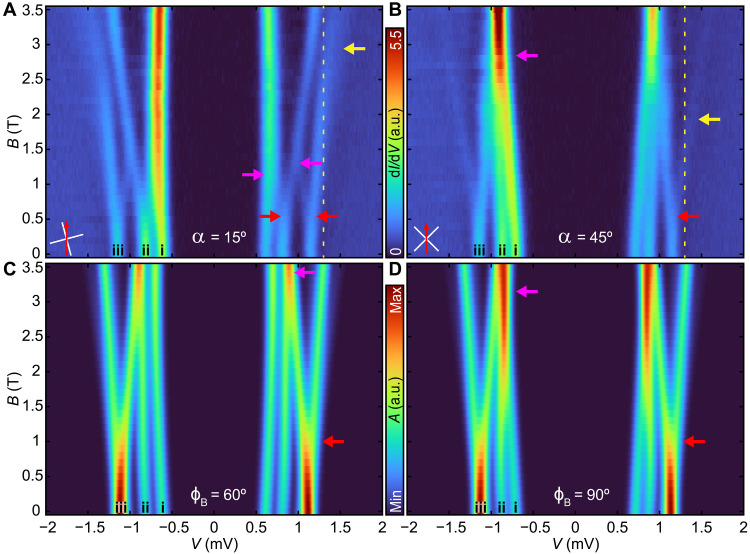
Transverse magnetic field dependence of the YSR excitations of MnPc2 given by coupled spins and two sites. (**A**) A false-color plot of the STS spectra of MnPc2(α = 15°) taken at *B*_∥_ in steps of Δ*B*_∥_ =0.1 T, up to *B*_∥_ = 3.5 T (*V*_S_ = 6 mV, *I*_t_ = 200 pA, *V*_mod_ = 20 μV), with α defined as in Fig. 1D. (**B**) A false-color plot of the STS spectra of MnPc2(α = 45°) taken at *B*_∥_ steps of Δ*B*_∥_ = 0.1 T, up to *B*_∥_ = 3.5 T. (**C**) Simulation of the YSR spectra using the two site model with parameters: *S*_1_ = 1, *S*_2_ = 1/2, *g*_1_ = 1.5, *g*_2_ = 2, *D*_1_ =−0.43, *E*_1_ =−0.1, *D*_2_ = *E*_2_ = 0, *J*_ex_ = 0.05, Δ_1,2_ = 1.3, *g*_SC_ = 2, *t* = 0, *J*_K_1__ = 2.14, *J*_K_2__ = 3.20, θ*_B_* = 90°, and ϕ*_B_* = 60°. (**D**) Same as (C) but with parameters: *S*_1_ = 1, *S*_2_ = 1/2, *g*_1_ = 1.5, *g*_2_ = 2, *D*_1_ =−0.35, *E*_1_ =−0.08, *D*_2_ = *E*_2_ = 0, *J*_ex_ = 0.05, Δ_1,2_ = 1.3, *g*_SC_ = 2, *t* = 0, *J*_K_1__ = 2.20, *J*_K_2__ = 3.23, θ*_B_* = 90°, and ϕ*_B_* = 90°. The various arrows were added to indicate particular states for discussion in the text.

Next, we turn to the MnPc2 molecules, which exhibited three YSR peaks in zero field. In contrast to MnPc1, their ligands are not aligned with a high-symmetry axis of the substrate. In the presence of a transverse magnetic field, we need to further distinguish the orientation of the MnPc2 molecules with respect to the B field. Here, we showcase two examples with different alignment angles α: MnPc2(α = 15°) and MnPc2(α = 45°), where α corresponds to the smallest angle of a molecular ligand axis (white dashed line in the inset of Fig. 1D) with respect to *B*_∥_ (red arrow) and α = 0 corresponds to *B*_∥_ being parallel to a ligand axis. We note that the orientation of *B*_∥_ remains fixed. In Fig. 4A, we illustrate the typical case for MnPc2(α = 15°). With increasing *B*_∥_, we observe a peak (i) that hardly shifts except for a small bowing with an inflection point toward the gap edge *B*_∥_ ≈ 2 T. It also increases both in its intensity and linewidth for *B*_∥_ > 0.5 T, where a peak splitting off from the second peak (ii) merges in. Not only that peak (ii) splits at about *B*_∥_ ≈ 0.5 T but also the outermost peak (iii) is split at this applied magnetic field (red arrows). At the intermediate field of *B*_∥_ ≈ 0.5 T, we thus detect in total five YSR excitations. From the different splitting of both peaks (ii) and (iii), we conclude that the excitations must involve states with different spin projections. Above *B*_∥_ ≈ 1 T, the inner branch of peak (ii) is not discernable anymore from resonance (i), while also the outer branch from (ii) and the inner one from (iii) merge (magenta arrows), reducing the number of observable YSR excitations to three. The other respective branches of peaks (ii) and (iii) move toward the gap edge with different slopes. The outer branch of peak (iii) crosses the coherence peak at *B*_∥_ ≈ 2.5 T (yellow dashed line) and continues outside the gap (yellow arrow). We note that when the branch of peak (iii) crosses the coherence peak, there are no observed sudden changes in the YSR spectra. Last, we do not observe a crossing or splitting of the merged peaks up to *B*_∥_≈ 4 T (fig. S9). This observation is contrary to the expectation of YSR excitations originating from different spin states that cross. It is also unexpected that the split-off branch from peak (ii) does not seem to continue after it merged with (iii).

In Fig. 4B, we illustrate the typical case for MnPc2(α = 45°). Peaks (i) and (ii) illustrate a nearly linear shift toward the gap edge with slightly different slopes, until *B*_∥_ ≈ 2.5 T. The outer peak (iii) remains relatively insensitive to a magnetic field up to *B*_∥_ ≈ 0.5 T and then subsequently splits (red arrow). The branch of peak (iii) that moves toward the gap center merges with the shifted peaks (i) and (ii) at *B*_∥_ ≈ 2.5 T (magenta arrow). As also seen for MnPc2(α = 15°), the other branch of peak (iii) crosses the location of the coherence peak (yellow dashed line) and persists outside of the gap in increasing values of *B*_∥_, with diminishing intensity. The crossing of the branch of peak (iii) occurs at lower values of *B*_∥_, compared to the merging of all the other observed states (magenta arrow). The merging of these peaks remains up to *B*_∥_= 4 T (fig. S9), leading to a broadening of the overall linewidth and increasing intensity. This is like the previous case, and this merging persists for Δ*B* ≈ 1.5 T without the observation of a crossing or splitting of states. We note that the intensity of the branch of state (iii) observed outside of the gap decreases.

The observation that both orientations of MnPc2 show distinctly different *B*_∥_ dependence illustrates the importance of transversal anisotropy and thus the quantum nature of the YSR excitations. For both orientations of MnPc2, we modeled the YSR excitations as two spin sites with *S*_1_ = 1, representing the spin on the Mn center, and *S*_2_ = 1/2, representing the spin on the ligand. As the molecules lay in different orientations with respect to the underlying Pb lattice, the electronic structure of MnPc molecule, including its crystal field and hybridization, may vary. This can be mimicked by considering different values of *D*, *E*, *J*_K_, and *S*, compared to MnPc1. In Fig. 4 (C and D), we illustrate MnPc2(ϕ_B_ = 60°) and MnPc2(ϕ_B_ = 90°), the modeled YSR spectra for MnPc2(α = 15°) and MnPc2(α = 45°), respectively, with ϕ_B_ defined as in Fig. 3B. Here, we consider a spin *S*_1_ = 1 with magnetic anisotropy coupled antiferromagnetically with a second spin *S*_2_ = 1/2 in a transverse magnetic field. We also display the corresponding energy-level diagrams (fig. S10) and transition amplitudes (fig. S11). The ground state of the system has two bound quasiparticles, with the YSR excitation creating an unbound quasiparticle on one of the two superconductor sites. Because of the impact of magnetic anisotropy on *S*_1_, the simulation yields three YSR excitations for *B*_∥_ = 0 T. In this case, the excitations labeled (ii) and (iii) are 2*E*_1_ and *D*_1_ + *E*_1_ higher in energy than (i), respectively, yielding three YSR excitations. Creating an unbound quasiparticle on site 2 involves four YSR excitations, transitioning to the singlet and triplet combination of *S*_1_ and *S*_2_ that are nearly degenerate (gray lines in fig. S10), as well as nearly degenerate with the highest excitation of *S*_1_ at *B*_∥_ = 0 T, yielding a total of three peaks at *B*_∥_ = 0 T.

Next, we consider *B*_∥_ > 0 T, for MnPc2(ϕ_B_ = 60°) in Fig. 4C. Excitation (i) first increases in energy and then becomes nearly constant for increasing *B*_∥_. Excitation (ii) follows the same trend as (i) with a slightly different slope. Peak (iii), which originates from both the third excitation of *S*_1_ and the YSR excitations from *S*_2_, splits linearly in energy as *B*_∥_ increases (red arrow) and crosses state (ii) at *B*_∥_ ≈ 3.5 T (magenta arrow). The number of observable YSR excitations thus changes from three (*B*_∥_ < 0.5 T) to four (0.5 T < *B*_∥_ < 3 T) and lastly to three (*B*_∥_ > 3 T). Last, we consider *B*_∥_ > 0 T, for MnPc2(ϕ_B_ = 90°) in Fig. 4D. Excitation (i) increases linearly in energy for increasing *B*_∥_, in contrast to the ϕ_B_ = 60° case. Excitation (ii) increases in energy and has an inflection (*B*_∥_ ≈ 1 T), resulting in a crossing of the peaks (i) and (ii) at *B*_∥_ ≈ 3 T (magenta arrow). To reproduce the one broad peak inside of the gap, we intentionally chose *g*_1_ < *g*_2_ such that this crossing occurs at a higher value of *B*_∥_and in tandem with the overlap of the YSR excitation stemming from the *S*_2_ (magenta arrow). Peak (iii) evolves the same as for MnPc2(ϕ_B_ = 60°). The number of observable YSR excitations thus changes from three (*B*_∥_ < 0.5 T) to four (0.5 T < *B*_∥_ < 3 T) and lastly to two (*B*_∥_ > 3 T).

While the model simulations of MnPc1 agree reasonably well with the experimental data, there are also clear differences in the simulated spectra and the experimental spectra with respect to MnPc2. There are three classes of observations that cannot be reproduced in the model for MnPc2: (a) certain nonlinear splitting, (b) the robustness of merged states in variable magnetic field, and (c) the physical nature of the observed out-of-gap excitations. As for (a), the model predicts a splitting of excitation (iii) that is symmetric in energy (red arrow), while in the experiment for MnPc2(α = 45°), we observe a nonlinear evolution of the split peaks, each of which has a different slope. In addition, the simulation does not capture the observed splitting of peak (ii) for MnPc2(α = 15°) around *B*_∥_ ≈ 0.5 T (red arrow). Last, neither the intensities of the YSR excitations at *B* = 0 T nor the evolution of the intensities is captured correctly in the simulation. This may be due to state-dependent and potentially magnetic field–dependent scattering as well as cotunneling through molecular orbitals (*27*), which are not considered. Concerning (b), on the basis of the model parameters, we cannot reproduce a merging of YSR excitations that remain merged for substantial changes in *B*_∥_ (magenta arrows). This is most notable for α = 45°, where around *B*_∥_ ≈ 2.5 T, these merges remain robust up to *B*_∥_ = 4 T (fig. S9). This merging suggests that the expected excitations cannot be treated independently once the states become degenerate. Last, (c), the model will not properly capture the continuation of the YSR excitations near the gap edge into the quasiparticle continuum (yellow arrows) and its effect on the YSR excitations that remain inside the gap. We further discuss these points below. However, these differences cannot be reproduced by considering substantially different values of the various model parameters, considering the constraint that both orientations of MnPc2 should stem from the same set of parameters. We note that we explored the effects of nonzero values of *t*. We generally found two effects at *B* = 0 T: (i) Eigenstates that were degenerate for *t* = 0 can become nondegenerate, and (ii) eigenstates become a mix of the two substrate sites, which both can result in additional transitions at *B* = 0 T. In addition, a nonzero *t* can also result in parallel YSR excitations as function of magnetic field. These effects are not observed (see fig. S12). Therefore, we set the term to zero.

The inability to capture the mentioned differences in the modeling of YSR excitations illustrates both the quantum nature of the problem and the potential need to consider typically neglected effects. As described above, the robust merging of the YSR excitations in variable magnetic field for both orientations cannot be completely described by exchange-coupled two-site excitations within the zero-bandwidth model [magenta arrows in Fig. 4 (A and B)]. Because of the nature of the YSR excitation, which links spin states of different spin projection, the Zeeman effect will lead to states crossing rather than merging. This can change in the case of a QPT or a spin transition of a given multiplet. An abrupt change in YSR excitations can occur near a QPT, which can result from either a change in fermion parity or a high/low-spin transition in the ground state. However, a QPT in fermion parity should be accompanied by one of the YSR excitations crossing the gap center which we do not observe. It has been observed that higher-order tunneling processes can lead to additional states near such a QPT (e.g., for *S* = 1/2) (*8*). However, it is not clear why higher-order processes would lead to a merging of states that persist for the observed ranges of *B*_∥_. In addition, we did not consider any nonequilibrium effects, like spin pumping. In the case of pumping, simplistically, additional states would be expected to emerge, similar to higher-order tunneling processes, and be dependent on, for example, *I*_t_. Moreover, the nature of the QPT is not well-defined when spin rotation symmetry is broken, due to transverse anisotropy or a transverse magnetic field. This is a result of the quantum nature of the problem, in which there is not a well-defined quantum number that describes the relevant spin states.

Last, the nature of the excitations that extend outside of the superconducting gap and their impact on the coincident YSR excitations that remain in the gap are also not known. Traditionally, these can be explained by inelastic excitations (*28*–*30*). Pair excitations out of the gap have been observed at zero field (*31*). However, in this case, it is unclear what the role of spin-orbit coupling combined with the applied magnetic field are on such excitations, as SU(2) symmetry is broken. These observations suggest that it may be important in future theoretical modeling to consider effects like renormalization, bath hopping, multi-electron processes, and cotunneling in these types of experiments.

## DISCUSSION

In conclusion, we track the evolution of YSR excitations between quantum states of a high-spin molecule on a superconductor using transversal magnetic field. This is based on a methodological approach that takes advantage of the highly enhanced upper critical transverse field of a BCS superconductor with high spin-orbit coupling when scaled to the two-dimensional limit. Taking advantage of this upper critical field enables one to study the spin-based behavior of individual impurities and their interactions in a variable magnetic field while remaining in the superconducting state. This approach is a pathway toward experimental methods that probe how spins evolve in magnetic field, such as electron spin resonance–STM (*32*), for example, at milli-Kelvin temperature (*33*, *34*), as well as ascertaining theoretical models about the role of YSR excitations on interacting spins (*35*, *36*). This hybrid approach which combines spins and ultrathin superconducting films is not limited to probing YSR excitations but can also be expanded to a number of other superconducting spintronic platforms, which are based on heterostructures of magnetic and nonmagnetic layers. We observe two different types of YSR excitations, MnPc1 and MnPc2, which are dictated by the orientation of the molecular ligands with respect to the underlying high symmetry axes of the Pb(111) surface. For each YSR excitation type, we observe behavior that, based on zero-bandwidth modeling, can be described by the interplay between one or multiple spins, magnetic anisotropy, and a transverse magnetic field. While the modeling qualitatively reproduces a number of the experimental features, there are also a number of unexpected experimental observations that are not readily reproduced by the conventional description of YSR excitations. For example, for MnPc2, we observed a merging of YSR excitations in applied magnetic field that persists. Likewise, we observed YSR excitations that shift out of the superconducting gap while concurrently observing YSR excitations in the gap, without any discontinuity. In addition to these unexpected observations, there is also a lack of observations that one might theoretically expect, such as a QPT in the spin projection or parity of the ground state which would be accompanied by a discontinuity in YSR excitations or any of the YSR excitations crossing the gap center. These observations motivate considering modeling that goes beyond, for example, the effects of cotunneling (*37*) on the atomic spin excitations, the presence of unexpected excitations and nontrivial electron-electron interactions, as well as relevant Kondo renormalization and spin pumping effects. Moreover, these observations may motivate phenomenological considerations of field-induced changes to the superconducting order parameter and its effect on the YSR excitations. These results present a pathway to study spin impurity models on superconductors in magnetic field and to explore the quantum nature of these excitations and their dynamics.

## MATERIALS AND METHODS

We used a home-built ultrahigh vacuum STM/STS facility, which operates at *T* = 30 mK, where a magnetic field can be applied either perpendicular as well as parallel to the sample surface (*34*, *38*). STS was taken using a lock-in method, with the modulation voltage applied to the sample. Si(111)−(7 × 7) was prepared by repetitive flashing to *T*~1450°C, as measured by a pyrometer aimed at the sample surface. Approximately 1 to 2 monolayer(s) (ML) of Ag was subsequently deposited at room temperature (RT) and post-annealed at *T*~575°C for 15 min to form the reconstruction Si(111)-Ag($\sqrt{3}\times \sqrt{3}$) (see fig. S1). Next, Pb was deposited, while the substrate was cooled on a liquid nitrogen cold stage (*T*~110 K). Last, the MnPc molecules were deposited on the substrate at RT, after which the substrate was transferred into a He flow-cryostat cooled transfer arm to stop the RT anneal time (4 min from cold stage to transfer arm) and transferred into the STM.

The prominent Moiré structure (*39*) and the resultant quantum well states (*40*, *41*) of the Pb films are detailed in fig. S1. We focused on Pb films that were 11 ML thick. At smaller energy scales, we observed a superconducting gap with an extracted gap value of Δ = 1.29 ± 0.01 mV using a normal metal tip, similar to previous reports using STS (*5*, *42*). The use of a normal tip combined with the measurement temperatures allows eliminating the use of a superconducting tip and the necessary field-dependent deconvolution of its states on the spectra. In fig. S2, we illustrate a typical spectrum of the Pb surface as a function of in-plane magnetic field (*B*_∥_) up to *B*_∥_ = 4 T, measured >4 nm away from any MnPc molecule center. MnPc molecules were laterally manipulated from the step edge of the Pb films and intentionally placed at various locations along the film surface. Lateral manipulation was performed with the following parameters (feedback closed, *V*_s_ = 10−200 mV, *I*_t_ = 1−2 nA).

The zero-bandwidth model used in this paper is composed terms for the substrate, YSR interaction, magnetic anisotropy, and exchange interaction all of which are described in detail in (*14*, *24*, *31*) as well as additional terms for the Zeeman energy of the spins sites and superconducting site

$$H=H_{\text{SC}}+H_{J_{K}}+H_{\text{MAE}}+H_{J_{\text{ex}}}+H_{\text{Zee}}$$

For the two spin and two superconducting sites model, this is

$$H_{\text{SC}}=\sum_{i=1}^{2}\Delta \left(c_{i,↑}^{†}c_{i,↓}^{†}+c_{i,↓}c_{i,↑}\right)-\sum_{\sigma =↑,↓}t\left(c_{1,\sigma }^{†}c_{2,\sigma }+c_{2,\sigma }^{†}c_{1,\sigma }\right)$$

$$H_{J_{K}}=\sum_{i=1}^{2}\;\sum_{\sigma =↑,↓}\;\sum_{\sigma \prime =↑,↓}\;c_{i,\sigma }^{†}\left(S_{i}\cdot {\hat{J}}_{K_{i}}\cdot s_{\sigma ,\sigma \prime }\right)c_{i,\sigma \prime }$$

$$H_{\text{MAE}}=\sum_{i=1}^{2}D_{i}S_{i,z}^{2}+E_{i}\left(S_{i,x}^{2}-S_{i,y}^{2}\right)$$

$$H_{J_{\text{ex}}}=S_{1}\cdot {\hat{J}}_{\text{ex}}\cdot S_{2}$$

$$H_{\text{Zee}}=-\sum_{i=1}^{2}g_{i}\mu_{B}B\cdot S_{i}-\sum_{i=1}^{2}\;\sum_{\sigma =↑,↓}\;\sum_{\sigma \prime =↑,↓}c_{i,\sigma }^{†}\left(g_{\text{SC}}\mu_{B}B\cdot s_{\sigma \sigma \prime }\right)c_{i,\sigma \prime }$$

Here, **S***_i_* is the vector of spin operators for spin site *i*, and $s=\frac{1}{2}\tau$ in terms of the vector of Pauli matrices τ. We note that without the Zeeman field on the superconducting sites, the *B*-field evolution is nonlinear (*13*). For the one spin, one superconducting site, *i* = 1, *t* = 0 and *H*_*J*_ex__ = 0.

To obtain the simulated YSR spectra, for each value of **B**, we calculate the transition coefficient for every excited state

$$a=\sum_{i=1}^{2}\sum_{\sigma =↑,↓}\sum_{\lambda }{|\langle \lambda |c_{i,\sigma }^{†}|\text{gs}\rangle |}^{2}+{|\langle \lambda |c_{i,\sigma }|\text{gs}\rangle |}^{2}$$

where ∣λ⟩ and ∣gs⟩ denote the excited and ground state, respectively, and subsequently plot two Gaussians at ±(*E*_λ_ *− E*_gs_) with amplitude *a*, and full width at half maximum = 0.1 mV to account for experimental broadening. Last, all Gaussian peaks are summed resulting in the simulated spectrum *A*(*E*, **B**). This modeling does not consider potential scattering, which will induce an asymmetry between the electron-like and hole-like excitations. There is also a double counting in our simulations for the transition coefficient *a* of the excitations (i.e., considering excitations with electrons and holes).
