# Supplementary material for: Quantifying the quantum nature of high-spin YSR excitations in transverse magnetic field
Source: Sci Adv. 2024 Oct 18;10(42):eadq0965. doi: 10.1126/sciadv.adq0965 (PMC11800828; doi:10.1126/sciadv.adq0965)
Supplement: Supplementary file 1 — Figs. S1 to S12 [file sciadv.adq0965_sm.pdf]

Science Advances
AAAS

# Supplementary Materials for

## Quantifying the quantum nature of high-spin YSR excitations in transverse magnetic field

Niels P. E. van Mullekom *et al.*

Corresponding author: Alexander A. Khajetoorians, a.khajetoorians@science.ru.nl

*Sci. Adv.* **10**, eadq0965 (2024)
DOI: 10.1126/sciadv.adq0965

**This PDF file includes:**

Figs. S1 to S12

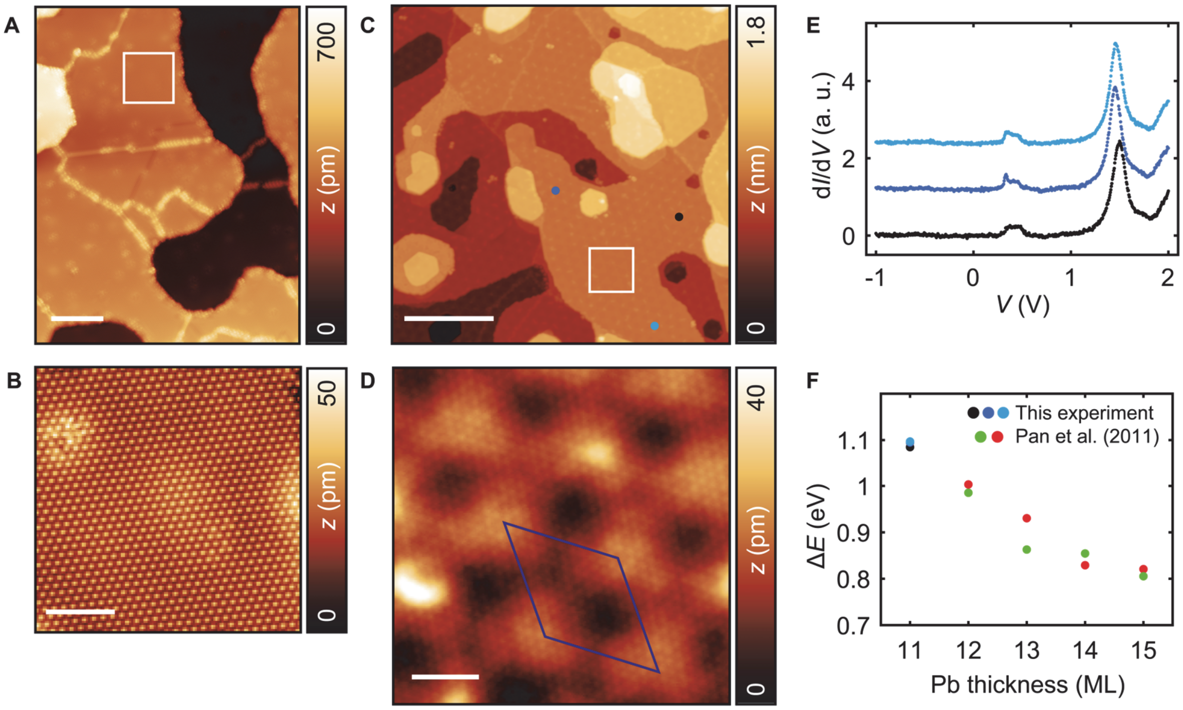


**Figure S1: Substrate characterization.** (**A**) Large-scale constant-current STM image of the reconstruction Si(111)-Ag($\sqrt{3} \times \sqrt{3}$) ($V_s = 1$ V, $I_t = 5$ pA, $T = 7$ K, scale bar: 20 nm). (**B**) Zoomed in constant-current STM image of the white square in (**A**) ($V_s = 1$ V, $I_t = 5$ pA, $T = 7$ K, scale bar: 5 nm). (**C**) constant-current STM image of a 11 ML Pb film on Si(111)-Ag($\sqrt{3} \times \sqrt{3}$) ($V_s = 90$ mV, $I_t = 5$ pA, $T = 30$ mK, scale bar: 20 nm). (**D**) constant-current STM image of the white square in (**c**) showing atomic resolution and the moiré-pattern. ($V_s = 3$ mV, $I_t = 6$ nA, $T = 30$ mK, scale bar: 2 nm). (**E**) STS measurements of the QWS at locations indicated in (**c**) by the colored dots, vertically offset for clarity ($V_s = 1$ V, $I_t = 10$ pA, $V_{mod} = 5$ mV, $T = 30$ mK). (**F**) Comparison of the difference in QWS energies in (**E**) with Ref. 42 for Pb films on Si(111)-Ag($\sqrt{3} \times \sqrt{3}$) (red dots) and Pb films on Si(111) (green dots).

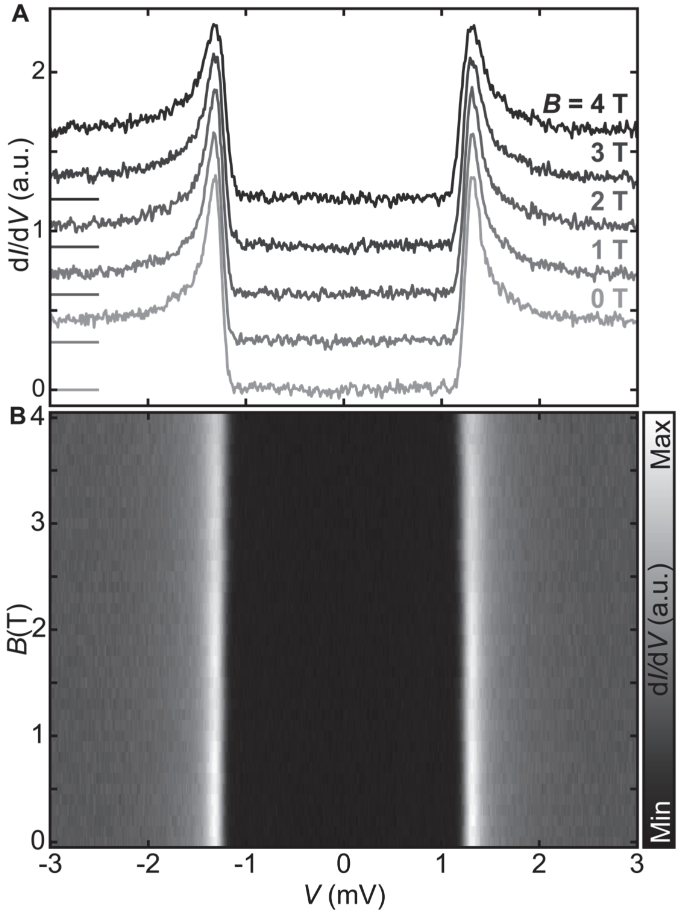


**Figure S2: SC gap spectra at variable magnetic field.** (**A**) Point spectra taken at transverse magnetic field strength indicated, vertically offset for clarity with zero indicated by horizontal lines. (**B**) a false-color plot of the STS spectra taken at $B_{\parallel}$ in steps of $\Delta B_{\parallel} = 0.1$ T, up to $B_{\parallel} = 4$ T. All data was measured with $V_S = 6$ mV, $I_t = 200$ pA, $V_{mod} = 20\ \mu$V, $T = 30$ mK and a W tip.

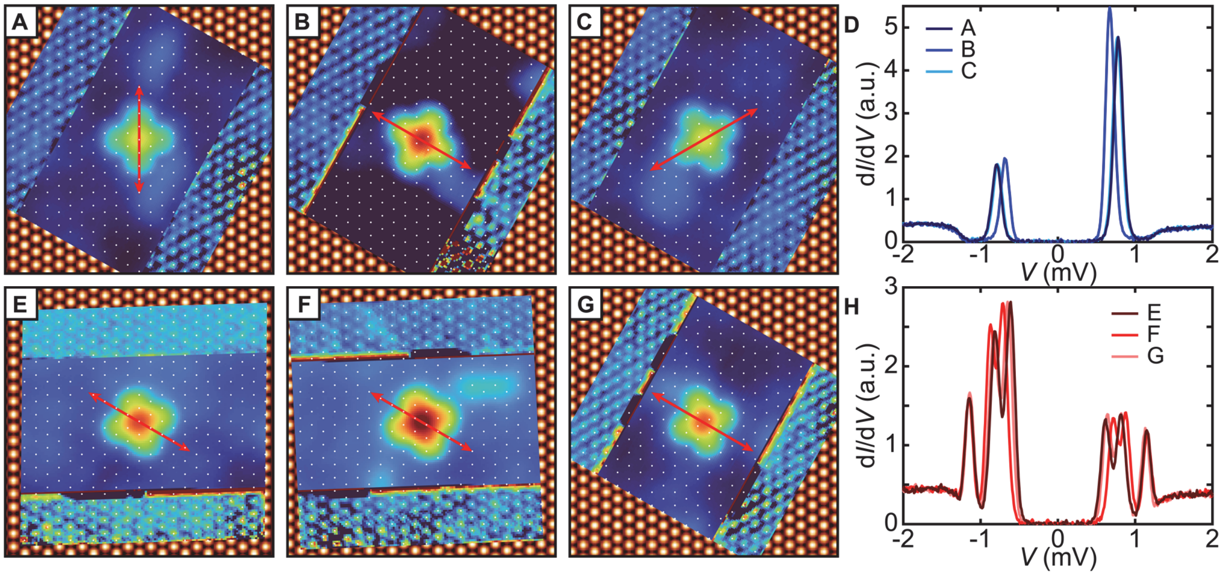


**Figure S3: Binding site analysis versus YSR spectrum.** (**A**-**C**) Processed constant-current STM- images of various MnPc1 on top of simulated Pb(111) lattice with atomic positions overlaid in white dots. The constant-current STM image was taken with two different parameter sets: for the atomic resolution away from the molecule ($V_S = 3$ mV, $I_t = 6$ nA) and with the molecule ($V_S = 90$ mV, $I_t = 20$ pA). The atomic resolution part was processed by separating it using a threshold, then flattened and scaled to enhance the contrast, and subsequently matched to the simulated lattice. Red arrows show ligand axis parallel to one of the high symmetry directions of Pb(111). (**D**) STS of MnPc shown in (**A**-**C**), showing typical MnPc1 YSR states. (**E-G**) Same as (**A**-**C**), but for MnPc2. The red arrows indicate the high symmetry direction of Pb(111) that bisects the ligand axes. (**H**) STS of MnPc shown in (**E**-**G**) showing typical MnPc2 YSR states. All spectra measured using $V_S = 6$ mV, $I_t = 200$ pA, $V_{mod} = 20\ \mu$V.

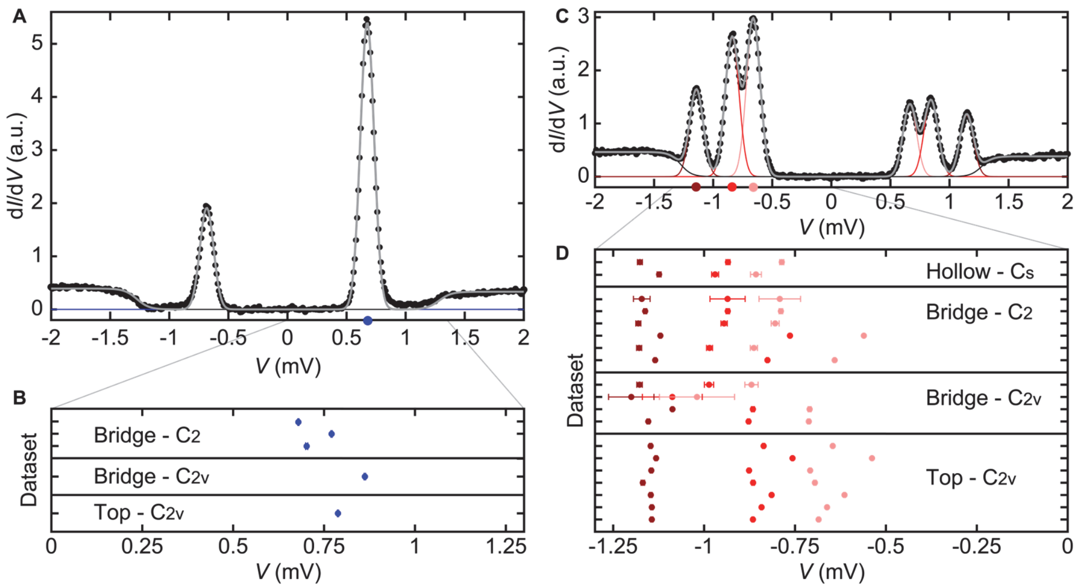


**Figure S4: Variance in YSR energies at zero magnetic field.** (**A**) Example fit (gray) of MnPc1 STS measurement (black dots), using a pair of Gaussians and a pair of broadened step functions symmetric around the Fermi energy. (**B**) Peak positions of 5 MnPc1 molecules for which the adsorption site and symmetry with respect to the Pb(111) lattice was determined (error bars from 95% confidence interval of the fit, smaller than icon size). (**C**) Example fit (gray) of MnPc2 STS measurement (black dots), using three pairs of Gaussians (red shades) and a pair of broadened step functions (black line) symmetric around the Fermi energy. (**D**) Peak positions of 19 MnPc2 molecules for which the adsorption site and symmetry with respect to the Pb(111) lattice was determined (error bars represent 95% confidence interval of the fit).

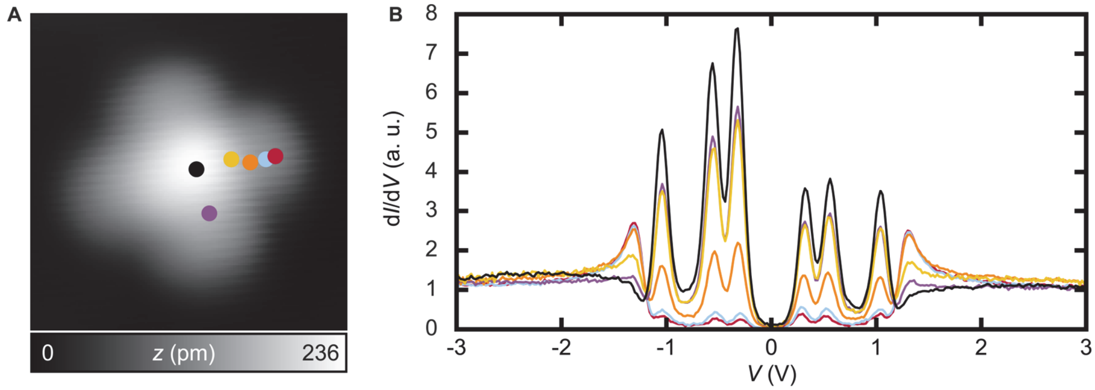


**Figure S5: Spatial dependence of the zero-field YSR excitations of MnPc2.** (**A**) Constant-current image of a MnP2 molecule ($V_S = 90$ mV, $I_t = 30$ pA). The colored circles refer to the locations where the spectra in (**B**) were taken. (**B**) STS of the YSR excitations of the molecule in (**A**), taken at indicated locations. The intensity of the excitations spatially vary, but all excitations are observable at all the probed locations at the same energies.

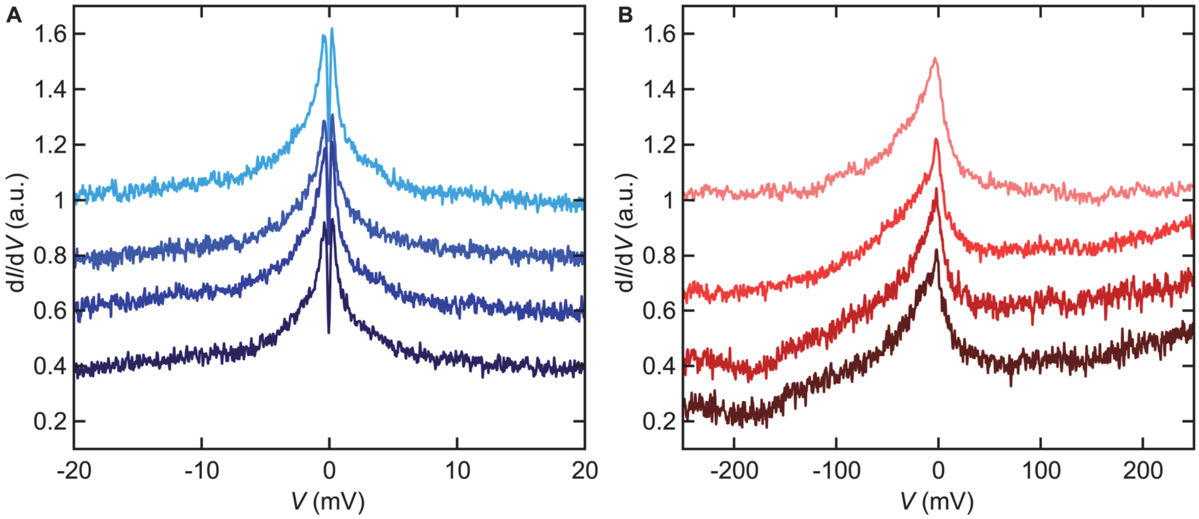


**Figure S6: Kondo spectra of MnPc.** The superconducting state of the Pb film is quenched by applying an out-of-plane magnetic field of $B_z = 0.5$ T. (**A**) STS of various MnPc1, showing a Zeeman-split Kondo resonance ($V_S = 20$ mV, $I_t = 200$ pA, $V_{mod} = 40$ $\mu$V). (**B**) STS of various MnPc2 featuring a broad Kondo resonance ($V_S = 250$ mV, $I_t = 100$ pA, $V_{mod} = 1$ mV).

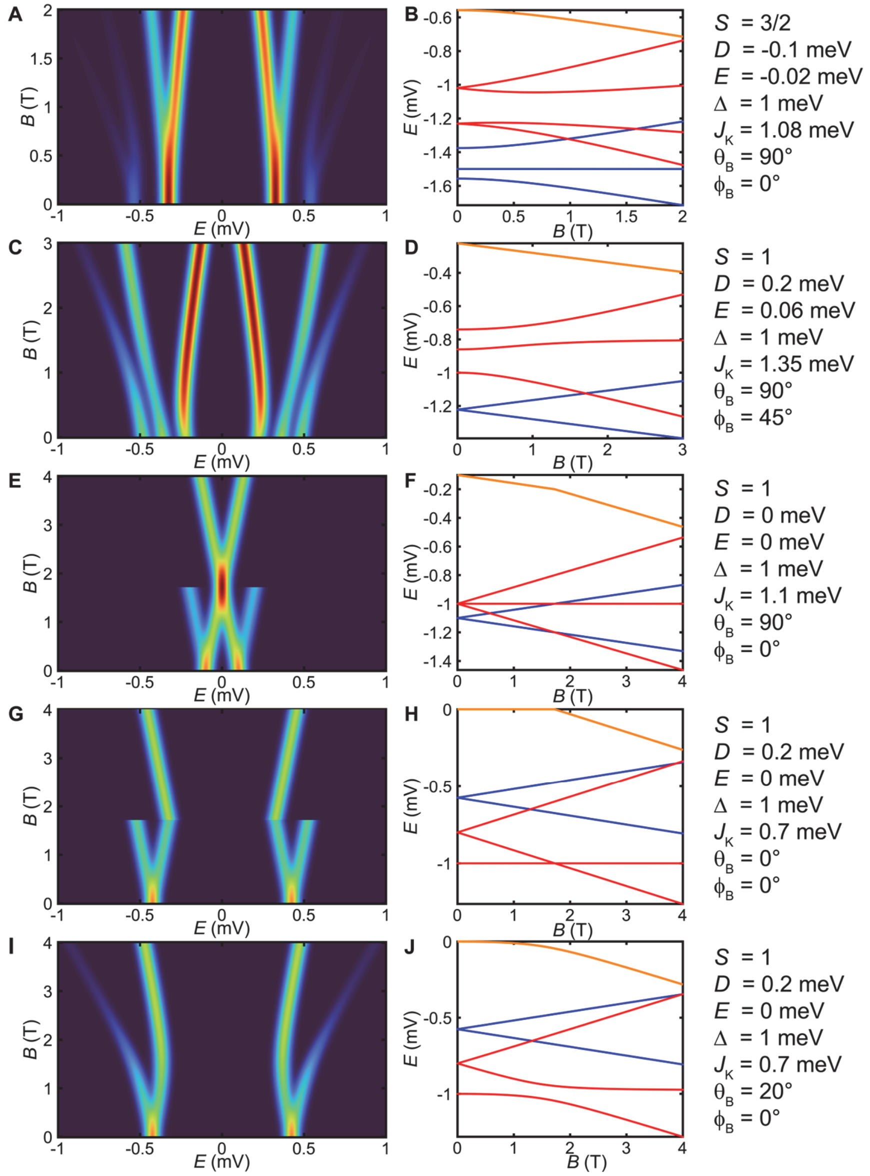


**Figure S7: Various calculations using the zero-bandwidth model, illustrating the various trends.** (**A**,**B**) An example of *B*-field dependent splitting of YSR excitations due to Kramer's degenerate excited states where the simulated spectra (left) and the energy level diagram (right) are illustrated. (**C**,**D**) An example of non-linear YSR excitations due to magnetic anisotropy where the simulated spectra (left) and the energy level diagram (right) are illustrated. (**E**,**F**) An example of a change in number of YSR excitations due to QPT in fermion parity where the simulated spectra (left) and the energy level diagram (right) are illustrated. (**G**,**H**) An example of a change in number of YSR excitations due to QPT in spin ground state where the simulated spectra (left) and the energy level diagram (right) are illustrated. (**I**,**J**) An example of broken spin rotation symmetry due to transverse magnetic field component where the simulated spectra (left) and the energy level diagram (right) are illustrated. In all energy level diagrams: red (blue) lines indicate states with $P = 1$ ($P = -1$), and orange lines correspond to $E_{gs} + \Delta$.

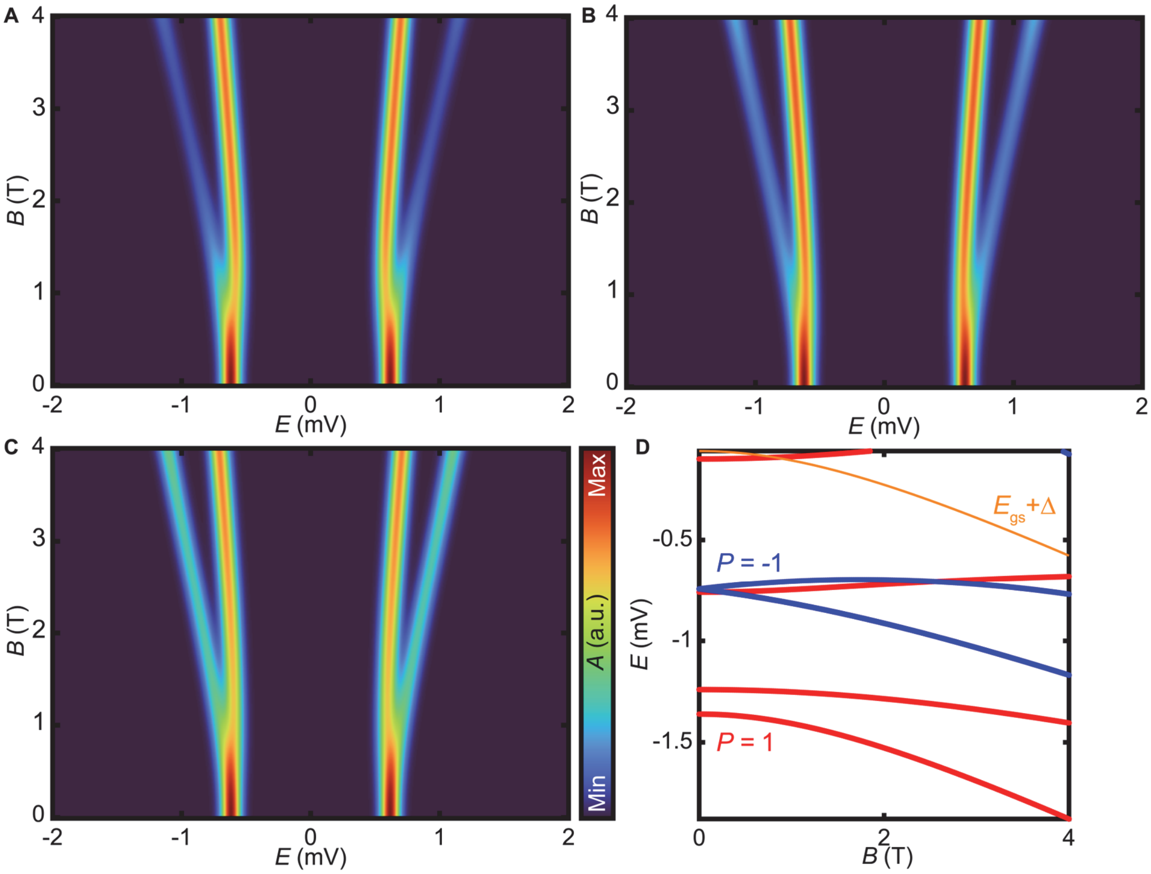


**Figure S8: Alternative YSR simulations for MnPc1.** (**A**) Reproduction of Fig. 3b plotted as reference. (**B**) Simulation with easy-plane anisotropy instead, using model parameters: $S = 1$, $g = 2$, $D = 0.3$ mV, $E = 0$ meV, $\Delta = 1.3$ mV, $J_K = 0.86$ mV, $\theta_B = 90°$, $\phi_B = 0°$. (**C**) Simulation with different total spin instead, using model parameters: $S = 2$, $g = 2$, $D = 0.3$ mV, $E = 0.08$ meV, $\Delta = 1.3$ mV, $g_{SC} = 2$, $J_K = 0.56$ mV, $\theta_B = 90°$ and $\phi_B = 0°$. (**D**) Partial energy level diagram of (**C**), showing lowest energy states up to the ground state, with the orange line being $E_{gs} + \Delta$.

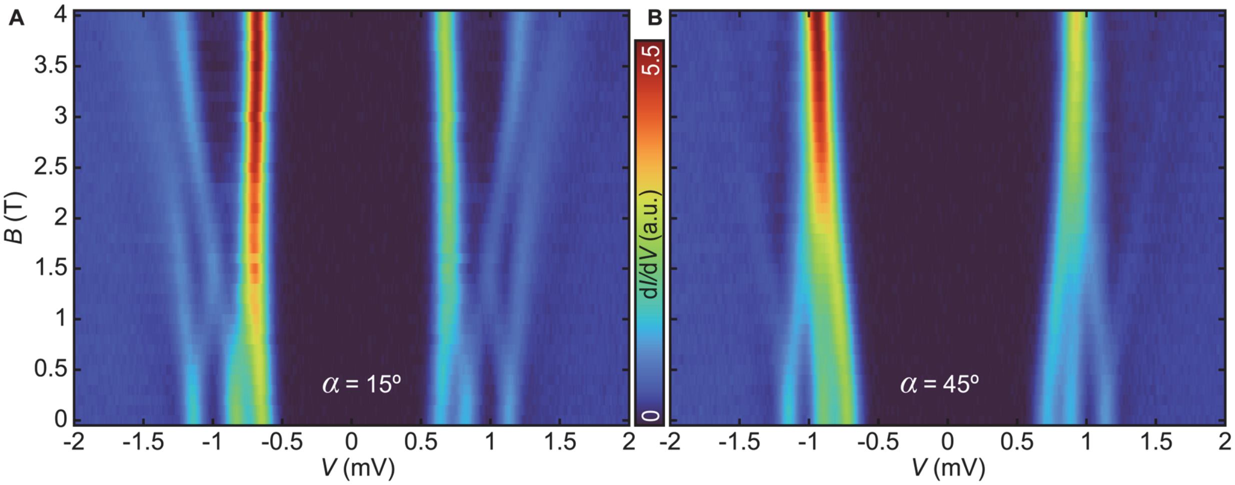


**Figure S9: Transverse magnetic field dependence of the YSR excitations of MnPc2.** (**A**) A false-color plot of the STS spectra of MnPc2($\alpha = 15°$) taken at $B_{\parallel}$ in steps of $\Delta B_{\parallel}$ =0.1 T , up to $B_{\parallel} = 4.0$ T ($V_S = 6$ mV, $I_t = 200$ pA, $V_{mod} = 20\ \mu$V). (**B**) A false-color plot of the STS spectra of MnPc2($\alpha = 45°$) taken at $B_{\parallel}$ steps of $\Delta B_{\parallel} = 0.1$ T, up to $B_{\parallel} = 4.0$ T.

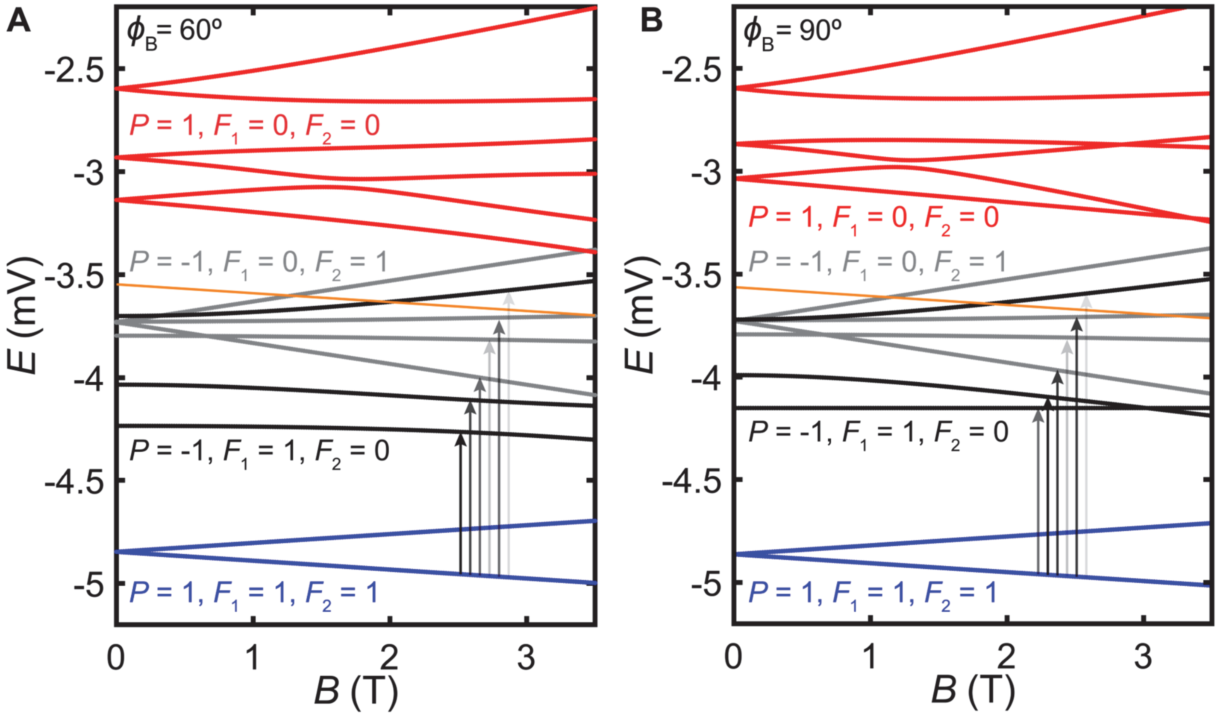


**Figure S10: Energy level diagram of MnPc2 YSR simulations in Fig. 4.** (**A**,**B**) The energy level diagram corresponding to Fig. 4C. and Fig. 4D, respectively. The orange line corresponds to the ground state energy $E_{gs} + \Delta$. $P$ is the fermion parity and $F_i$ is the number of bound quasiparticles on SC site $i$. Arrows are drawn for possible YSR excitations with their intensities schematically representing the transition amplitudes.

**A**

| a, λ1 ≡ \|gs> to: | Transverse magnetic field (T) | | | | | | | |
|---|---|---|---|---|---|---|---|---|
| | 0 | 0.5 | 1 | 1.5 | 2 | 2.5 | 3 | 3.5 |
| λ2 | 0 | 0 | 0 | 0 | 0 | 0 | 0 | 0 |
| λ3 | 0.385 | 0.398 | 0.412 | 0.430 | 0.452 | 0.479 | 0.509 | 0.538 |
| λ4 | 0.338 | 0.395 | 0.438 | 0.463 | 0.471 | 0.465 | 0.448 | 0.429 |
| λ5 | 0.258 | 0.204 | 0.495 | 0.495 | 0.495 | 0.495 | 0.495 | 0.495 |
| λ6 | 0.248 | 0.495 | 0.157 | 0.119 | 0.091 | 0.070 | 0.055 | 0.044 |
| λ7 | 0.247 | 0.301 | 0.348 | 0.386 | 0.414 | 0.435 | 0.450 | 0.461 |
| λ8 | 0.247 | 0.208 | 0.150 | 0.107 | 0.077 | 0.056 | 0.042 | 0.032 |
| λ9 | 0.277 | 0 | 0 | 0 | 0 | 0 | 0 | 0 |
| λ10 | 0 | 0 | 0 | 0 | 0 | 0 | 0 | 0 |
| λ11 | 0 | 0 | 0 | 0 | 0 | 0 | 0 | 0 |
| λ12 | 0 | 0 | 0 | 0 | 0 | 0 | 0 | 0 |
| λ13 | 0 | 0 | 0 | 0 | 0 | 0 | 0 | 0 |
| λ14 | 0 | 0 | 0 | 0 | 0 | 0 | 0 | 0 |
| λ15 | 0 | 0 | 0 | 0 | 0 | 0 | 0 | 0 |

**B**

| a, λ1 ≡ \|gs> to: | Transverse magnetic field (T) | | | | | | | |
|---|---|---|---|---|---|---|---|---|
| | 0 | 0.5 | 1 | 1.5 | 2 | 2.5 | 3 | 3.5 |
| λ2 | 0 | 0 | 0 | 0 | 0 | 0 | 0 | 0 |
| λ3 | 0.374 | 0.374 | 0.374 | 0.374 | 0.374 | 0.374 | 0.374 | 0.607 |
| λ4 | 0.338 | 0.432 | 0.502 | 0.547 | 0.574 | 0.590 | 0.600 | 0.374 |
| λ5 | 0.258 | 0.205 | 0.495 | 0.495 | 0.495 | 0.495 | 0.495 | 0.495 |
| λ6 | 0.248 | 0.495 | 0.158 | 0.120 | 0.092 | 0.072 | 0.056 | 0.045 |
| λ7 | 0.247 | 0.300 | 0.347 | 0.384 | 0.413 | 0.433 | 0.448 | 0.460 |
| λ8 | 0.247 | 0.195 | 0.124 | 0.080 | 0.053 | 0.036 | 0.026 | 0.019 |
| λ9 | 0.288 | 0 | 0 | 0 | 0 | 0 | 0 | 0 |
| λ10 | 0 | 0 | 0 | 0 | 0 | 0 | 0 | 0 |
| λ11 | 0 | 0 | 0 | 0 | 0 | 0 | 0 | 0 |
| λ12 | 0 | 0 | 0 | 0 | 0 | 0 | 0 | 0 |
| λ13 | 0 | 0 | 0 | 0 | 0 | 0 | 0 | 0 |
| λ14 | 0 | 0 | 0 | 0 | 0 | 0 | 0 | 0 |
| λ15 | 0 | 0 | 0 | 0 | 0 | 0 | 0 | 0 |

**Figure S11: Tables of transition amplitudes for the calculated YSR excitations for MnPc2.** (**A**, **B**) Transition amplitudes from ground state to shown excited states in the calculates YSR states shown in Fig. 4(**A**, **B**) and Fig S8(**A**, **B**), respectively. Note that the eigenstates $\lambda 1 - \lambda 15$ are sorted from lowest to highest energy for value of the magnetic field.

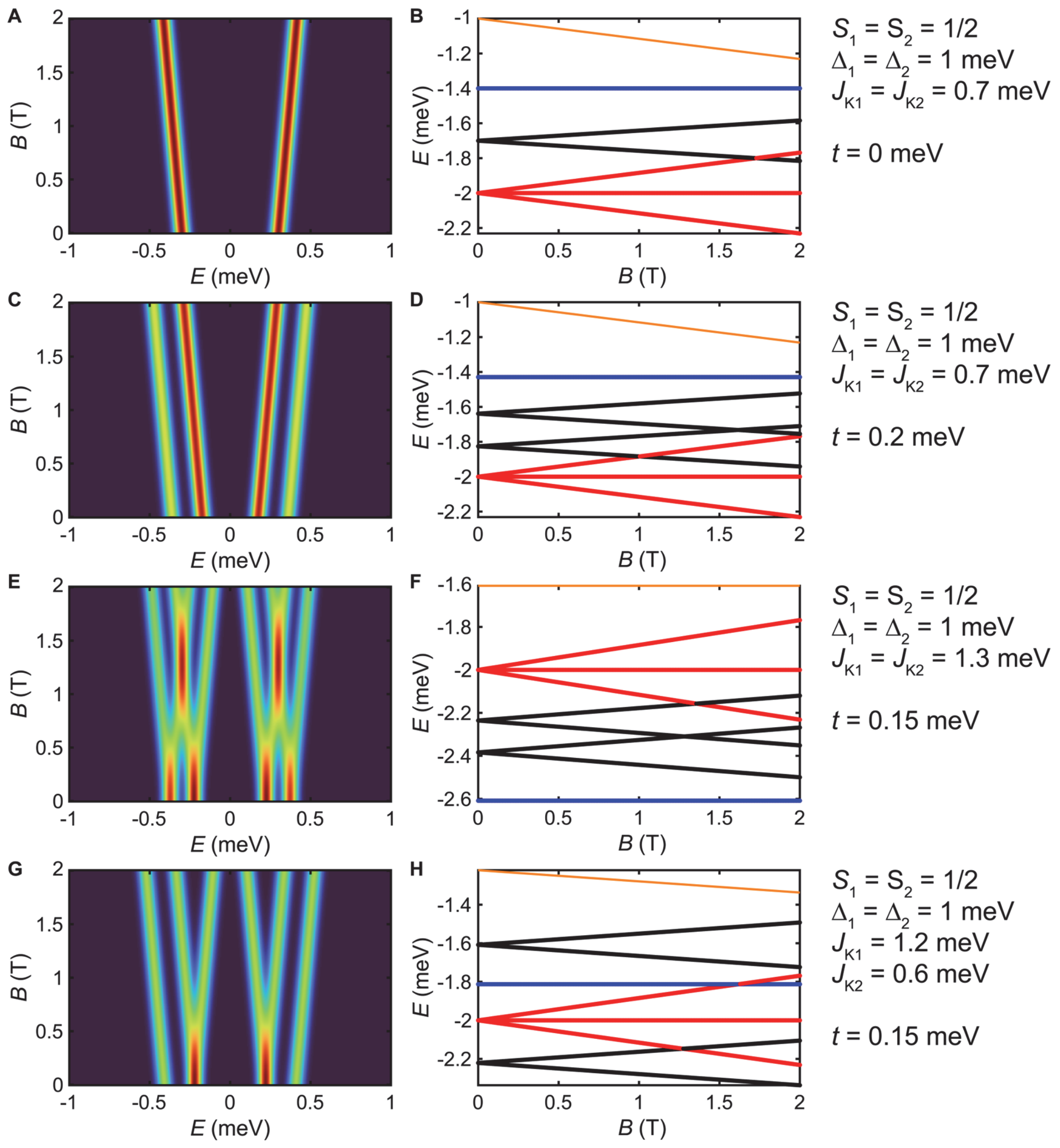


**Figure S12: Influence of hopping parameter *t* on YSR excitations.** (**A**, **B**) Simulated YSR spectrum and associated energy level diagram for two unbound spin 1/2 impurities as function of magnetic field, without hopping ($t = 0$). The color of the lines in the energy level diagram is an approximation for the amount of bound quasiparticles (red = 0, black = 1, blue = 2), and the relevant simulation parameters are noted on the right. (**C**, **D**) Same as (**A**, **B**) but with hopping $t = 0.2$ meV. The YSR is now degenerate at $B = 0$ T, and its field dependence is duplicated. (**E**, **F**) Similar to (**C**, **D**) but for two bound quasiparticles. Note that the YSR excitations cross each other. (**G**, **H**) Case where one quasiparticle is bound and one is unbound due to different Kondo exchange parameters $J_{K_1} > \Delta_{1,2} > J_{K_2}$.
